# Relationship Between High Organ Donation Rates and COVID-19 Vaccination Coverage

**DOI:** 10.3389/fpubh.2022.855051

**Published:** 2022-04-11

**Authors:** Yusuke Inoue

**Affiliations:** Department of Public Policy, The Institute of Medical Science, The University of Tokyo, Tokyo, Japan

**Keywords:** vaccination, COVID-19, organ donation, trust, social capital, medical professionals, solidarity, OECD countries

## Abstract

**Background:**

Besides attaining the goal of self-protection, the rollout of vaccination programs also encourages altruistic practices. Therefore, the progress in vaccination against coronavirus disease (COVID-19) in each country may be related to the prevalence of cooperative and altruistic practices in health care. I hypothesized that in countries where organ donation is popular, individuals would exhibit a greater tendency to become vaccinated.

**Methods:**

I examined the correlation between the level of progress of COVID-19 vaccination and the status of organ donation just before the pandemic in Organization for Economic Co-operation and Development (OECD) countries. Publicly available statistical information on the progress of immunization and organ donation was used. Univariate and multivariate analyses were conducted to examine common drivers of immunization and organ donation.

**Results:**

In OECD countries, progress in vaccination was found to be significantly correlated with the status of organ donation in each country. This relationship was stable after the summer (September 1: Pearson's *r* = 0.442, October 1: 0.457, November 1: 0.366). The results of the univariate and multivariate analyses showed that high trust in medical professionals was significantly correlated with both the “progress of vaccinations” and “organ donations.”

**Conclusions:**

Progress in COVID-19 vaccination and organ donation status for transplantation have similar trends, and both may involve people's trust in medical personnel and public health systems. Similar to the efforts to obtain organ donors, governments around the world need to take further steps to ensure that vaccination programs are supported by people's trust and sense of solidarity.

## Introduction

By the end of October 2021, coronavirus disease (COVID-19), caused by severe acute respiratory syndrome coronavirus 2 (SARS-CoV-2), had infected over 200 million individuals and caused more than 5 million deaths; however, there is still no end in sight to the pandemic (1). Nevertheless, in 2021, in addition to pre-existing restrictions, several types of vaccines that may control the spread of infection to a certain degree were introduced. Although there are concerns about the persistence of efficacy (2) and the need to be vigilant regarding new viral mutations, there are high hopes for the effectiveness of these vaccines in controlling infection.

Many developed nations of the world have made great progress in vaccinating their populations against SARS-CoV-2. However, a detailed examination of worldwide data, even when limited to data of the Organization for Economic Co-operation and Development (OECD) member nations, shows that there are major differences in the progress of vaccination programs in each country. For example, according to statistics published in “COVID-19 Data Explorer” (Our World in Data) as of May 2021 (3), Israel (59.9%), followed by the United Kingdom (UK, 45.9%), Chile (36.2%), and the United States of America (USA, 33.1%) were the countries with the highest percentage of people who had received at least one COVID-19 vaccine dose. However, as the summer passed, many of these countries were overtaken by other countries (mainly European countries) that had increased their vaccination efforts. As of October 1, 2021, Portugal was in the lead (88.2%), followed by Iceland (82.1%), Chile (81.1%), and Spain (81.7%) (Table 1).

**Table 1:** The percentage (%) prevalence of COVID-19 vaccination (top eight OECD member nations).

	**April 2021**	**June 2021**	**August 2021**	**September 2021**	**October 2021**
1	Israel (59.9)	Israel (62.2)	Iceland (78.4)	Portugal (85.6)	Portugal (88.2)
2	United Kingdom (45.9)	Canada (58.1)	Denmark (72.2)	Iceland (81.4)	Iceland (82.1)
3	Chile (36.2)	United Kingdom (58.0)	Chile (72.1)	Spain (78.3)	Chile (81.1)
4	United States (33.1)	Chile (56.6)	The Netherlands (71.5)	Denmark (75.7)	Spain (80.7)
5	Hungary (22.4)	Hungary (54.1)	Canada (71.2)	Chile (75.1)	South Korea (77.2)
6	Finland (16.8)	United States (51.9)	Portugal (70.1)	Ireland (74.4)	Denmark (76.6)
7	Estonia (15.9)	Iceland (51.7)	Belgium (69.5)	Canada (73.4)	Canada (76.5)
8	Iceland (14.4)	Finland (44.6)	Spain (68.8)	The Netherlands (73.2)	Norway (76.4)

*OECD, Organization for Economic Co-operation and Development; COVID-19, coronavirus disease; N.B. People who were fully or partly vaccinated against COVID-19 in the population were included in the study*.

*Data source: Our World in Data (Available online at: https://ourworldindata.org/covid-vaccinations) “Share of people vaccinated against COVID-19”*.

What certainly lies in the background of the progress made by these countries is each country's independent initiatives to obtain and supply the vaccine, but I believe that this cannot fully explain their success. Given that there was little experience and high risks associated with the vaccine, people were not vaccinated simply because their government encouraged them to do so (4). Thus, there is a need to consider why people in these countries responded to their governments' calls to get vaccinated.

Besides attaining the goal of self-protection, the rollout of vaccination programs also encourages altruistic practices—e.g., lowering the risk of infection in vulnerable populations or maintaining the functioning of the healthcare system. According to Pywell, among altruistic medical practices, the tense relationship between individuals' self-determination and social interests tends to surface, especially in relation to vaccination and organ donation programs (5). When considering COVID-19 vaccination, vaccination progress may be smoother in countries that have successfully coordinated the relationship between the two. Thus, I hypothesized that in countries where organ donation is popular, individuals would exhibit a tendency to get vaccinated. To the best of my knowledge, there are no reports focusing on a predilection toward organ donation when considering the progress in COVID-19 vaccination.

## Materials and Methods

Using publicly available statistical information, I examined the correlation between progress in immunization against SARS-CoV-2 infection and the status of organ donation for transplantation just before the pandemic in OECD countries.

The progress in vaccination was based on data from the COVID-19 Data Explorer (Our World in Data), as mentioned above. Individuals who were fully or partly vaccinated against COVID-19 were included in the study. Donated organ transplant data were obtained from The Global Observatory on Donation and Transplantation (http://www.transplant-observatory.org/). The number of transplants per one million population in all countries in 2019, just before the pandemic, was used in the analysis.

Univariate (Pearson's correlation coefficients) and multivariate analyses (multiple linear regression) were conducted to examine organ donation and common drivers of immunization, using publicly available data [“trust in medical professionals” (6), “trust in the government” (7), “social solidarity” (8), “GDP” (9)]. All analyses were conducted using SPSS Statistics version 27.0 (IBM Corp., Armonk, NY, USA).

## Results

**Figure 1:**
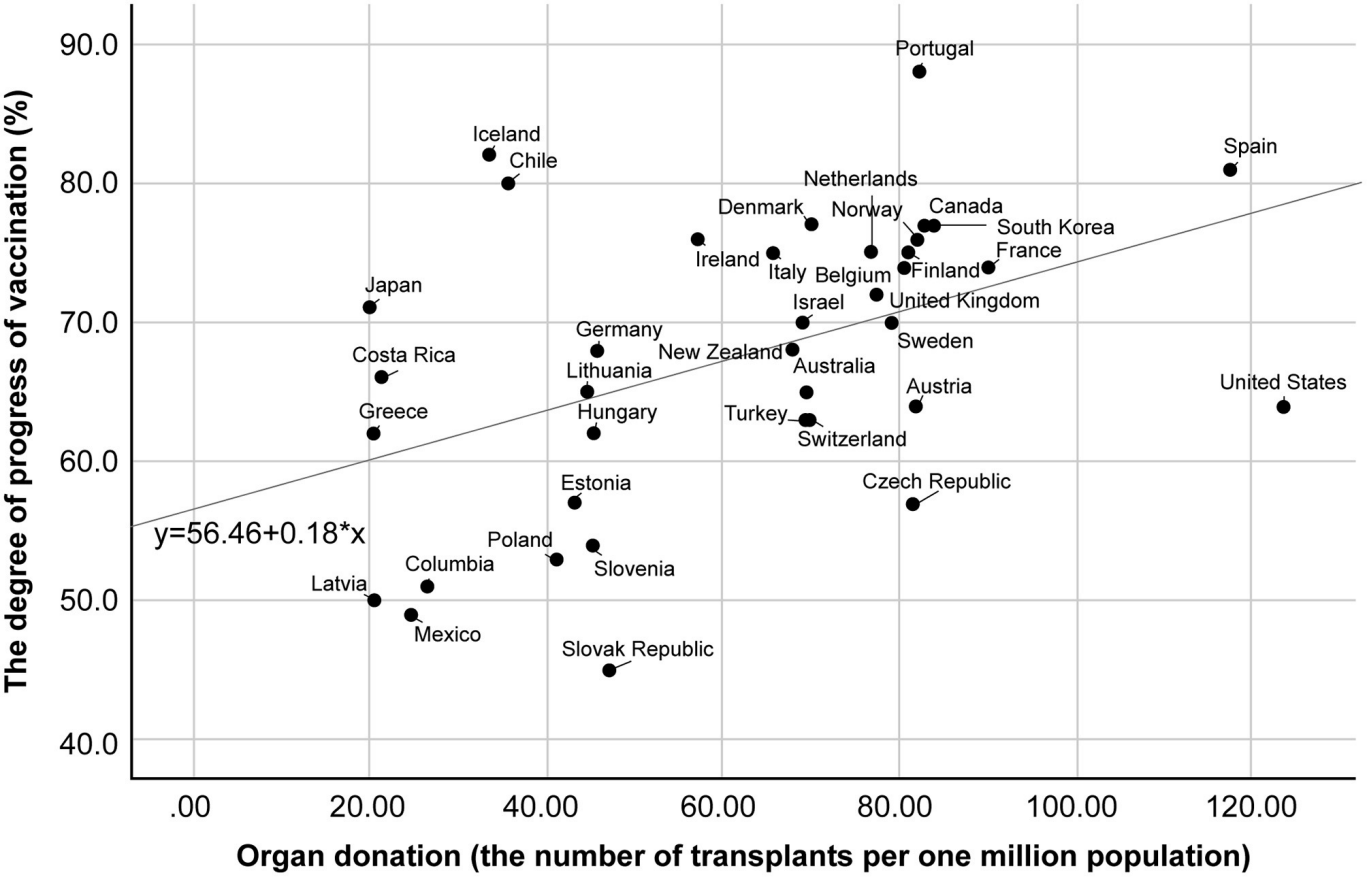
Correlation between COVID-19 vaccination progress and the number of organ donations. COVID-19, coronavirus disease.

I compared the progress in COVID-19 vaccination with the actual number of donated organ transplants in 38 OECD countries. Figure 1 shows the relationship between the number of organ donations (number of donations per million population) and the progress in COVID-19 vaccination (percentage of people vaccinated in the population). The correlation was loose, but significant, and it persisted during the summer and fall of 2021 when the vaccination program was in full swing among developed countries (September 1: Pearson's *r* = 0.442, October 1: 0.457, November 1: 0.366). Spain and France are typical cases where organ donation has been relatively active and vaccination has proceeded well. In contrast, both organ donation and vaccination have been sluggish in Greece. Meanwhile, the United States, has had a sluggish vaccination rollout compared to its thriving organ donation program. Iceland, Chile, and Japan, conversely, are experiencing a slump in organ donation, but are doing relatively well in vaccination.

Both the number of organ donations (Pearson's *r* = 0.438, *p* < 0.01) and the prevalence of COVID-19 vaccination (Pearson's *r* = 0.592, *p* < 0.01) in these OECD member nations showed a loose correlation with trust in medical professionals (see Supplementary Table 1A). The results of the multivariate analysis also showed that high trust in medical professionals was significantly correlated with both the “progress of vaccinations” and “organ donations” (see Supplementary Table 1B).

In this study, other factors that have been considered to have a significant association with the progress in COVID-19 vaccination were also examined. The results of the univariate analysis replicated these relationships. With the exception of trust in the government, these factors also showed significant relationships with the progress in COVID-19 vaccination. However, multivariate analysis did not reproduce these relationships (see Supplementary Table 1B).

It is also worth noting that in the univariate analysis, many of the factors found to be associated with the progress in COVID-19 vaccination also showed significant relationships with willingness to donate organs. However, these factors, with the exception of trust in the medical profession, did not show significant relationships in multivariate analysis.

The value of the adjusted coefficient of determination (adjusted R-squared) was mild, which indicates that factors other than those examined in this study may also have a significant impact. Nevertheless, the consistently high level of trust in health care providers was stable and deserves mention as a strong candidate for a common factor deeply related to the progress in COVID-19 vaccination and organ donation.

## Discussion

Several investigations regarding the factors promoting COVID-19 vaccinations have been conducted. The following factors have been reported to have an effect on the progress in COVID-19 vaccination: educational background and gender (10, 11); old age (10–12); country income level (13); transparency of government activities (14); numbers of infected and deceased individuals (15); degree of vaccine acceptance (16) [e.g., use of the seasonal influenza vaccine (17, 18)]; digital infrastructure (19); and trust in governments (11, 15, 18, 20), physicians (18), and scientists (21, 22).

Although conducting a detailed investigation of the correlation between COVID-19 vaccinations and the number of organ donations is not straightforward, I believe that the two share common facilitating factors. However, whether there is a cause-and-effect relationship is unclear.

One hypothesis is that trust in specialists plays an important role in both activities. The results of the analysis showed that high trust in medical professionals was significantly correlated with both the “progress of vaccinations” and “organ donations.”

Medical professionals have advanced medical knowledge and skills, use the latest medical techniques, and are simultaneously in positions in which they spread knowledge and use these skills and techniques. If trust in medical professionals is low, it is unlikely that the number of people who are willing to receive the novel vaccines will increase. In addition, those who appreciate their country's health care system will be more likely to get vaccinated, if they understand that COVID-19 places an enormous burden on the system.

Another hypothesis is that this can be explained by shared understanding of the role of the individual in public health. Organ donations and vaccinations are activities groups engage in, and they test the strength of the links between trust in existing governing institutions and the degree of group consciousness (23, 24). If a sense of solidarity is awakened in times of crisis (25), then societies that are more supportive of organ donation may tend to be more united in the face of public health disasters. Naturally, as mentioned above, the fact that medical professionals enjoy a high degree of trust in the community can be a factor that contributes greatly to community participation in vaccination programs and to the expressed desire of many in the community to donate organs. Moreover, based on the correlation between univariates, the development of vaccinations is related to a high degree of trust in governments (Pearson's *r* = 0.451, *p* < 0.01). At the same time, organ donation is associated with a strong sense of solidarity in society (Pearson's *r* = 0.497, *p* < 0.05) (see Supplementary Table 1A). However, probably due to the small sample size and the correlation between variables, there was no clear relationship between the two in the multivariate analysis. More detailed studies are required in the future (see Supplementary Table 1B).

The above discussion is based on the premise that organ donation and vaccination have much in common as public health efforts. However, I also have to consider that there are meaningful differences between organ donations and vaccinations. For example, vaccination is mainly intended for healthy people, and individuals are expected to act on their own initiative. On the other hand, in the case of organ donation, especially post-mortem, the actions of others and institutional efforts are essential. Regarding organ donation situations, many countries have adopted so-called “opt-out” mechanisms in which the individual does not necessarily give explicit consent (26). Past studies have indicated that social factors, such as relationships with family members (27) and active intervention involving the community (28, 29), can relate to the progress in organ donation. Whereas, individual concerns about vaccination are about the risk and efficacy of the vaccine, concerns about the fairness and the sustainability of the system are likely to come to the fore in the case of organ donation (30). Despite these differences, there remains an important commonality between vaccination and organ donation; they are essentially public health issues that cannot be addressed by one person alone, and the role entrusted to health care providers and the health care system is of great importance. I have not found any issues that conflict with our results, and this understanding deserves consideration as a noteworthy finding.

This study had several limitations. Although the study scope was limited to OECD member nations, cultural and social backgrounds surrounding transplantation medicine, and the timing of securing vaccinations were different for each country. In addition, some statistical analyses were limited by the small sample size. Furthermore, other factors that strongly influence both COVID-19 vaccination and organ donations may not have been fully considered. Nevertheless, it is noteworthy that there are clear differences in the speed of COVID-19 vaccination between countries, and these differences may be related to fundamental public health and medical care factors.

Currently, COVID-19 vaccination programs are progressing around the world. However, in many countries, vaccination numbers are decreasing after reaching a peak in the summer. Particularly, in countries that were among the first to introduce vaccination programs (e.g., USA, Israel, UK), ~30% of their populations remain unvaccinated and the number of people receiving vaccinations has not increased since the summer. An important issue is how to approach those who refuse to be vaccinated. One subject of debate is whether to make COVID-19 vaccination obligatory. Additionally, given that a lot is expected from medical professionals, it is recommended that they get vaccinated. Naturally, consideration must be paid to the concerns of individual medical professionals, but if their vaccination will be effective in increasing appreciation for, and trust in, COVID-19 vaccines, then it may serve as an incentive for the general public to consider getting vaccinated.

Because of concerns regarding the sustainability of the effectiveness of COVID-19 vaccinations, even in countries where large numbers of people have already been vaccinated, there is a perceived need for COVID-19 booster vaccinations. In the interests of community and public health, it is important that booster vaccination programs are undertaken. Even in such cases, the presence of medical professionals who are attentive to people's concerns and interests will be critical.

To conclude, progress in vaccination against COVID-19 infections in OECD countries in 2021 is significantly associated with people's trust in medical personnel and public health systems, common to organ donation. Although there is no end to the pandemic in sight, people's sense of cooperation and high level of interest are not necessarily endless. Similar to the efforts to obtain organ donors, governments around the world need to laud and show appreciation for the cooperation people have shown so far, and to take further steps to ensure that vaccination programs are supported by people's trust and sense of solidarity. There is also a need to consider and address anxiety and distrust of medical professionals not only in terms of individual physician–patient relationships, but also as a public health issue.

## Data Availability Statement

Publicly available datasets were analyzed in this study. This data can be found here: All data used in this paper are publicly available only. All sources of these data have been acknowledged in the text and in the Supplementary File.

## Author Contributions

YI conceptualized the original study and prepared the article draft.

## Funding

This work was supported by the Research on Ethical, Legal and Social Issues (ELSI) of New Coronavirus Infectious Diseases COVID-19 grant of the Ministry of Health, Labour and Welfare Research Project for the Promotion of Emerging Re-emerging Infectious Diseases and Immunization Policy [20HA2011].

## Conflict of Interest

The author declares that the research was conducted in the absence of any commercial or financial relationships that could be construed as a potential conflict of interest.

## Publisher's Note

All claims expressed in this article are solely those of the authors and do not necessarily represent those of their affiliated organizations, or those of the publisher, the editors and the reviewers. Any product that may be evaluated in this article, or claim that may be made by its manufacturer, is not guaranteed or endorsed by the publisher.

## Abbreviations

COVID-19: coronavirus disease 2019
SARS-CoV-2: severe acute respiratory syndrome coronavirus 2
OECD: Organization for Economic Co-operation and Development.
