# Supplementary material for: Relationship Between High Organ Donation Rates and COVID-19 Vaccination Coverage
Source: Front Public Health. 2022 Apr 11;10:855051. doi: 10.3389/fpubh.2022.855051 (PMC9038079; doi:10.3389/fpubh.2022.855051)
Supplement: Supplementary file 1 [file Table_1.pdf]

Supplementary file 1

Table: Examination of the common factors that propel vaccinations and organ donations

a) Results of the Pearson‘s correlation coefficients

	1)	2)	3)	4)	5)	6)
1) Progress of vaccinations[(1)]	-					
Pearson‘s correlation coefficients						
(Number of valid variables)						
2) Trust in medical professionals[(2)]		-				
Pearson‘s correlation coefficients	0.438**					
(Number of valid variables)	(38)					
3) Trust in the government[(3)]			-			
Pearson‘s correlation coefficients	0.451**	0.586**				
(Number of valid variables)	(37)	(37)				
4) Social solidarity[(4)]				-		
Pearson‘s correlation coefficients	0.232	0.734**	0.643**			

(Number of valid variables)	(26)	(26)	(25)			
5) GDP(5)					-	
Pearson‘s correlation coefficients	0.348*	0.502**	0.663**	0.604**		
(Number of valid variables)	(38)	(38)	(37)	(26)		
6) Organ donations(6)						-
Pearson‘s correlation coefficients	0.457**	0.592**	0.267	0.497*	0.453**	
(Number of valid variables)	(37)	(37)	(36)	(25)	(37)	

**p<0.01, *p<0.05.

b) Results of the multiple linear regression

Response variable	Explanatory variable	Unstandardized Coefficients		Standardized Coefficients	t value	Adjusted R Square
		B	St. Error	β		
Progress of vaccinations(1)	Trust in medical professionals(2)**	0.432	0.126	0.801	3.416	0.412
	Trust in the government(3)	0.231	0.171	0.358	1.352	

	Social solidarity[4]*	-106.407	43.391	-0.636	-2.452	
	GDP[5]	0.037	0.105	0.087	0.349	
	(Const.)**	121.410	30.409		3.993	
Organ donations[6]	Trust in medical professionals[2]**	0.948	0.259	0.812	3.655	0.486
	Trust in the government[3]	-0.395	0.350	-0.264	-1.129	
	Social solidarity[4]	15.754	95.876	0.044	0.170	
	GDP[5]	0.098	0.262	0.087	0.373	
	(Const.)	14.336	64.583		0.222	

$^{**}p<0.01$, $^{*}p<0.05$.

(1) The degree of COVID-19 vaccination progress utilizes data as of October 1, 2021. Data source: Our World in Data (https://ourworldindata.org/covid-vaccinations) “Share of people vaccinated against COVID-19,” which indicates the percentage of vaccinated people in the population. Those both fully and partly vaccinated against COVID-19 were included in the study.

(2) Gallup. “Confidence and trust in health professionals and hospitals” (Chart 3.6 & Q11E in Appendix D: Dataset and crosstabs for all countries). Wellcome Global Monitor 2018 (https://wellcome.org/reports/wellcome-global-monitor/2018).

(3) OECD. “Trust in Government” (10. Core Government Results). Government at a Glance (2019). https://doi.org/10.1787/8ccf5c38-en.

(4) “Solidarity index by country” (Table 5). Sanz García M, Caselles A, Micó Ruiz JC, Soler Fernández D. Development of the Happiness Index in a country. Systems & Design: Beyond Processes and Thinking (2016) 807–818. http://dx.doi.org/10.4995/IFDP.2016.3096.

(5) International Monetary Fund. GDP (nominal) per capita. "World Economic Outlook Database, April 2021".

(6) Organ transplant data shows the number of transplants per one million population in all countries in 2019. Global Observatory on Donation and Transplantation(http://www.transplant-observatory.org/) was utilized.
